# Operative versus conservative treatment of apophyseal avulsion fractures of the pelvis in the adolescents: a systematical review with meta-analysis of clinical outcome and return to sports

**DOI:** 10.1186/s12891-017-1527-z

**Published:** 2017-04-19

**Authors:** H. Eberbach, L. Hohloch, M.J. Feucht, L. Konstantinidis, N.P. Südkamp, J. Zwingmann

**Affiliations:** Department of Orthopaedic and Trauma Surgery, University of Freiburg Medical Center, Hugstetter Straße 55, Freiburg, 79106 Germany

**Keywords:** Avulsion fractures, Pelvis, Treatment, Outcome, Return to sports

## Abstract

**Background:**

Avulsion fractures of the pelvic apophyses typically occur in adolescent athletes due to a sudden strong muscle contraction while growth plates are still open. The main goals of this systematic review with meta-analysis were to summarize the evidence on clinical outcome and determine the rate of return to sports after conservative versus operative treatment of avulsion fractures of the pelvis.

**Methods:**

A systematic search of the Ovid database was performed in December 2016 to identify all published articles reporting outcome and return to preinjury sport-level after conservative or operative treatment of avulsion fractures of the pelvis in adolescent patients. Included studies were abstracted regarding study characteristics, patient demographics and outcome measures. The methodological quality of the studies was assessed with the Coleman Methodology Score (CMS).

**Results:**

Fourteen studies with a total of 596 patients met the inclusion criteria. The mean patient age was 14.3 ± 0.6 years and 75.5% of patients were male. Affected were the anterior inferior iliac spine (33.2%), ischial tuberosity (29.7%), anterior superior iliac spine (27.9%), iliac crest (6.7%) lesser trochanter (1.8%) and superior corner of the pubic symphysis (1.2%). Mean follow-up was 12.4 ± 11.7 months and most of the patients underwent a conservative treatment (89.6%). The overall success rate was higher in the patients receiving surgery (88%) compared to the patients receiving conservative treatment (79%) (*p =* 0,09). The rate of return to sports was 80% in conservative and 92% in operative treated patients (*p =* 0,03). Overall, the methodological quality of the included studies was low, with a mean CMS of 41.2.

**Conclusion:**

On the basis of the present meta-analysis, the overall success and return to sports rate was higher in the patients receiving surgery. Especially in patients with fragment displacement greater 15 mm and high functional demands, surgical treatment should be considered.

## Background

Apophyseal avulsion fractures of the pelvis are injuries that typically occur in adolescent athletes [1]. At this age the secondary ossification at the apophyses coincides with the hormonally induced strengthening of the muscles [2]. Sudden large tension forces can be applied through the musculotendinous units due to forceful concentric muscle contraction or passive lengthening of the muscle especially during sporting activities. Because the cartilaginous growth plates at the apophyses of the adolescents are more prone to trauma than the musculotendinous units, they may fail resulting in a avulsion fracture of the pelvis [3]. Patients usually report a crack in the pelvic region during an activity with a sudden onset of pain [4, 5]. The pain is more severe during activity and decreases with rest. Clinical examination reveals local tenderness, limitation of motion, and swelling [6, 7]. The apophyses may fracture at the rectus femoris insertion at the anterior inferior iliac spine (AIIS), the hamstrings insertion at the ischial tuberosity (IT), the sartorius insertion at the anterior superior iliac spine (ASIS), the tensor fasciae latae insertion on the iliac crest (IC), the iliopsoas insertion on the lesser trochanter (LT), or the rectus abdominis insertion at the superior corner of the pubic symphysis (SCPS) [8–10]. Historically, most of the pelvic avulsion fractures have been treated nonoperatively including analgetics, bed rest, immobilization of the affected muscle group, and physical rehabilitation [11]. However, controversy persists which fractures and patients may benefit from operative treatment [5, 12]. The Grade of fracture displacement and the physical demands of the athlete can be important factors in the decision process for or against surgery [5, 11]. Long-term and sport-specific outcomes of these fractures have not been well studied and there is little data on the incidence of complications like non-union, heterotrophic ossifications, neurological sequelae, wound infections, persistent pain or functional restrictions. The available evidence appears unsatisfactory. The purpose of this systematic review with meta-analysis was to assess and summarize the patient demographics, epidemiology, mechanism of injury, clinical outcome, and return to sports to give support in the decision-making process regarding therapy in adolescent patients with avulsion fractures of the pelvis.

## Methods

### Literature search

A systematic search of the Ovid database (including MEDLINE, PreMEDLINE, EBM Reviews, Cochrane Database, Cancerlit, CINAHL, and EMBASE) was performed on December 4, 2016 to identify studies reporting clinical and sport-specific outcome after apophyseal fractures of the pelvis. This study was performed in accordance with the PRISMA (Preferred Reporting Items for Systematic Reviews and Meta-analysis) guidelines [13] The search strategy comprised the Boolean operators (AND; OR) that combined the following terms in the title and abstract fields: apophysis, apophyseal, fracture, injury, avulsion, avulsion*, pelvis and pelvic.

No limits were set on the date of publication. The inclusion criteria were specified in advance: Clinical studies of all kind and all levels of evidence published in English or German language, reporting clinical outcome after operative or conservative treatment of apophyseal avulsion fractures of the pelvis in the adolescent athlete, published online or in print in a peer-reviewed journal, including the results of at least 5 or more patients (till the age of 18 years, both sexes). Exclusion criteria were as follows: None-English or None-German-language studies, articles that were off-topic, study collectives with concomitant injuries (as pelvic ring fractures) or procedures, in-vitro or animal studies, radiologic or diagnostic studies, epidemiologic studies, and other types of articles such as technical notes, or narrative reviews.

Two reviewers (HE and LH) independently screened all articles for relevance by title and abstract according to the defined in- and exclusion criteria. If no abstract was available, the full-text article was obtained to assess the relevance of the study. The full-text of all articles not excluded during the initial screening process was obtained and reviewed by the same two reviewers for possible inclusion in the study. Any disagreement on article eligibility was resolved through discussion. To ensure that all studies were recorded, references within included studies and all review studies were cross-referenced for inclusion if missed by the initial search.

### Data extraction

All studies that met the inclusion criteria were abstracted regarding study characteristics, patient demographics, location and cause of injury, treatment protocols, and surgical technique. Data was collected by one reviewer (HE) in an Excel extraction form and verified by a second reviewer (LH). Any disagreement that arose was resolved by consensus between both reviewers. Study characteristics of interest included author names, year of publication, number of patients at final follow-up, length of follow-up, and the quality assessment by the Coleman Methology Score. Patient demographics included sex and the mean age of the patients. Outcomes of interest were the clinical outcome at follow-up, the complication rate (in particular non-union, heterotrophic ossifications, neurological sequelae, wound infections, persistent pain or functional restrictions like limited range of motion) and the proportion of patients returning to sports. Excellent outcome was specified as a reported excellent outcome and the absence of complications at follow-up. Return to sport was defined as the rate of patients returning to sports to the preinjury sport-level at follow-up.

### Quality assessment

The methodological quality of the included studies was evaluated with the modified Coleman Methodology Score. This score is widely used in systematic reviews and meta-analyses to assess the methodology of clinical studies by using 10 specific quantitative and qualitative criteria: study size, mean follow-up, number of surgical procedures, type of study, diagnostic certainty, description of surgical procedure, postoperative rehabilitation, outcome measures, outcome assessment, and selection process [14–16]. The final score ranges from 0 to 100, with a score of 100 indicating the highest study quality [17]. If the score is greater than 85, the study is designated as excellent, between 70 and 84 good, from 50 to 69 moderate, and below 50 poor [18]. In addition, all included studies were assessed according to the level of evidence defined by the Oxford Centre for Evidence Based Medicine [19].

### Statistics

The data was analyzed using established statistical software (Excel Version 14.3.9, IBM SPSS Statistics for Macintosh version 22.0 and R-Project Version 3.2.4, package ‘metaprop’, by G. Schwarzer, University of Freiburg). Descriptive statistics and calculation of frequency weighted means and standard deviations were used to report study characteristics, patient demographics, and outcomes. Pairwise comparisons were evaluated using the chi-squared test. A *p*-value of <0.05 was considered significant for all comparisons. Meta-analyses were performed to calculate the overall pooled rate of clinical outcome, complications, and return to sports after avulsion fractures of the pelvis. The random effects model was used for the meta-analysis. The result of the meta-analysis was presented as a forrest plot and heterogeneity was calculated according to the method of Higgins et al. and is expressed as I^2.^ The value of I^2^ ranges from 0% expressing complete consistency to 100% expressing complete inconsistency of the data [20].

## Results

### Study characteristics and Quality Assessment

**Fig. 1:**
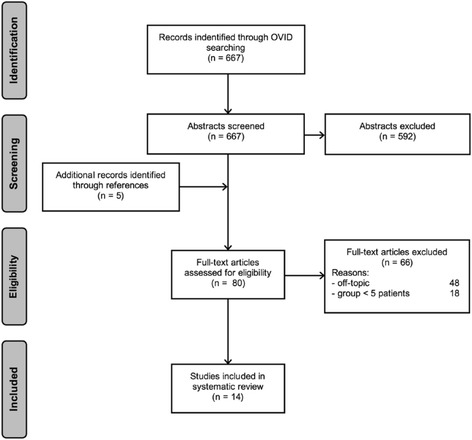
Search strategy. Flowchart of search strategy in accordance with PRISMA (Preferred Reporting Items for Systematic Reviews and Meta-analyses) guidelines

Out of 667 articles identified through the initial search and 5 additional records detected through the reference lists, a total of 14 studies met the inclusion criteria [3, 5, 7, 8, 10–12, 21–27]. The flow diagram according to PRISMA guidelines summarizes the selection protocol (Fig. 1). The patient demographics and study characteristics are shown in Table 1. The mean modified Coleman Methodology Score was 41.2 ± 9.0 (range, 28–52). The methodological quality of included studies was therefore low. All of the 14 included studies were Level IV evidence. The corresponding values of each study are shown in Table 1.

**Table 1:** Patient demographics and study characteristics

Lead Author	Year	n	Mean Follow-up, mo	CMS	CMS Level of evidence	Sex, F/M, n	Mean Age, y	ST/CT total
Schuett	2015	228	10	42	IV	55/173	14.4	2/226
Serbest	2015	5	2,5	37	IV	1/4	13.6	0/5
Kautzner	2014	23	12	42	IV	4/19	15.1	13/10
Li	2014	10	11	56	IV	N/A	14.6	10/0
Pogliacomi	2014	9	48	52	IV	2/7	16	9/0
Uzun	2014	9	26	45	IV	0/9	14	0/9
Ferlic	2013	13	>24	52	IV	1/12	15	5/8
Gidwani	2007	6	N/A	34	IV	1/5	15.2	4/2
Kosanovic	2002	6	N/A	28	IV	0/6	16	6/0
Rossi	2001	203	N/A	41	IV	64/139	13.8	3/200
Linni	2000	15	12	52	IV	3/12	12.5	0/15
Sundar	1994	22	44	54	IV	8/14	13.8	0/22
Metzmaker	1985	27	N/A	46	IV	5/22	15.8	0/27
Fernbach	1981	20	N/A	37	IV	2/18	N/A	0/20
Summary	-	596	12.4 ± 11.7^a^	41.2 ± 9.0^a^	IV	146/443	14.3 ± 0.6^a^	52/544

*n* total patients, *N/A* not available, *CMS* Coleman Methology Score, *ST* surgical treatment, *CT* conservative treatment

^a^frequency-weighted mean ± SD

### Patient characteristics

In total, 596 patients were enrolled in this meta-analysis. The number of patients in the included studies ranged from 5 to 228, with a mean of 43 patients per study. There were 440 males, representing 75.5% of the total study population. The mean age of the patients ranged from 12.5 to 16.0 years, with a mean age across all studies of 14.3 ± 0.6 years (Table 1).

### Locations and cause of injury

**Fig. 2:**
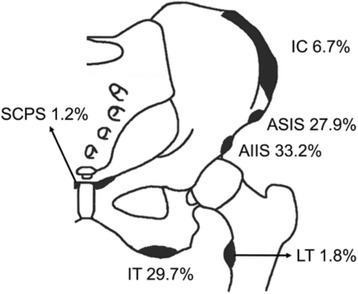
Locations of injury. Schematic diagram showing the various pelvic avulsion fractures and their relative frequencies. AIIS, anterior inferior iliac spine; IT, ischial tuberosity; ASIS, anterior superior iliac spine; IC, iliac crest; LT, lesser trochanter; SCPS, superior corner of the pubic symphysis

The most common sites of avulsions were the anterior inferior iliac spine (AIIS) in 33.2%, ischial tuberosity (IT) in 29.7%, anterior superior iliac spine (ASIS) in 27.9%, iliac crest (IC) in 6.7%, lesser trochanter (LT) in 1.8%, and superior corner of the pubic symphysis (SCPS) in 1.2% of the cases (Fig. 2). The sport disciplines primarily predisposing to AIIS and IT avulsion fractures were ball sports (70% and 45%, respectively), to ASIS avulsion fractures ball sport and athletics in equal parts (both 46%), to IC avulsion fractures athletics (63%), and to LT and SCPS avulsion fractures ball sports (with 67% and 86%, respectively).

### Treatment protocols

For conservative treatment, partial weight bearing was allowed at an average of 0.1 weeks (range, 0–3 weeks) and full weight bearing after 4.9 weeks post incidentally (range, 3–6 weeks). Return to normal activity including sports was allowed on average at 3.1 months postoperatively (range, 2–6 months).

Operative treatment was performed with screws in 76%, k-wires in 15%, and plates in 9%. Regarding operative treatment, partial weight bearing was allowed instantly in all studies and full weight bearing after a mean of 4.5 weeks postoperatively (range, 1–8 weeks). Return to normal activity including sports was allowed on average at 2.4 months postoperatively (range, 1–6 months).

### Clinical outcome

**Fig. 3:**
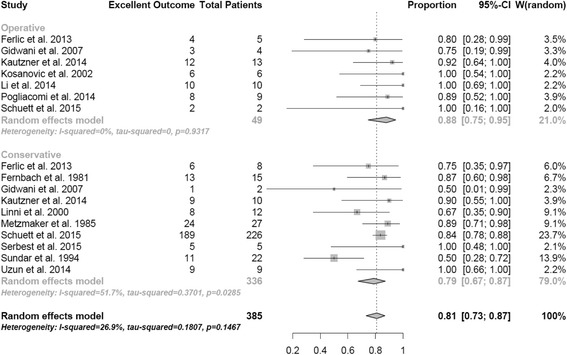
Outcome at follow-up. Random effect model representing the proportion of patients with excellent outcome at follow-up. The horizontal lines extending out of the squares represent the 95% CI surrounding the best estimate. The diamond represents the weighted pool with confidence intervals

**Fig. 4:**
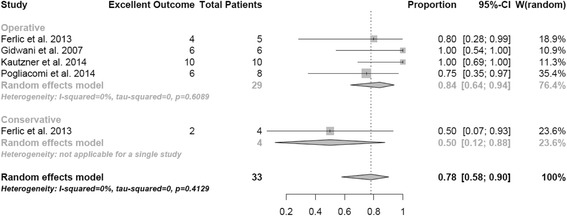
Outcome at follow-up after treatment of more than 15 mm dislocated apophyseal fractures. Random effect model representing the proportion of patients with excellent outcome after treatment of more than 15 mm dislocated apophyseal fractures. The horizontal lines extending out of the squares represent the 95% CI surrounding the best estimate. The diamond represents the weighted pool with confidence intervals

The rate of excellent outcome was higher (*p =* 0,09) in patients receiving surgery with 88% (95% CI, 0.75-0.95) compared to the rate of patients receiving conservative treatment with 79% (95% CI, 0.67-0.87) (Fig. 3). Comparing avulsion fractures with a displacement of more than 15 mm the difference became significant (*p =* 0,04) with excellent results in 84% of the operatively and only 50% in the conservatively treated patients (Fig. 4).

### Complications

**Fig. 5:**
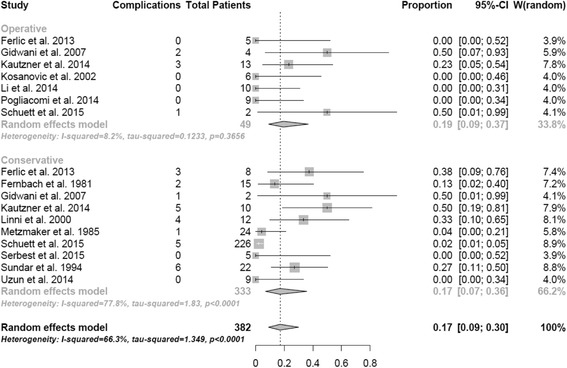
Complications at follow-up. Random effect model representing the proportion of complications. The horizontal lines extending out of the squares represent the 95% CI surrounding the best estimate. The diamond represents the weighted pool with confidence intervals

Complications (in particular non-union, heterotrophic ossifications, neurological sequelae, wound infections, persistent pain or functional restrictions) were assessed in 13 studies and 382 patients (64.1% of study group) as shown in Fig. 5. The overall complication rate in this meta-analysis according to the random effects model was 17.0% (95% CI, 0.09-0.30). The complication rate was discretly lower in the conservative subgroup compared to the operative subgroup (17.0% vs. 19.0%). This difference was not significant (*p =* 0,34). The rate of non-unions was lower in the operatively treated (0%) compared to the conservatively treated patients (2.4%), whereas there were more heterotopic ossifications in the operative subgroup (8.2%) than in the conservative subgroup (2.4%). Neurological complications and functional restrictions were present in both subgroups in about 2% of the cases.

### Return to sports

**Fig. 6:**
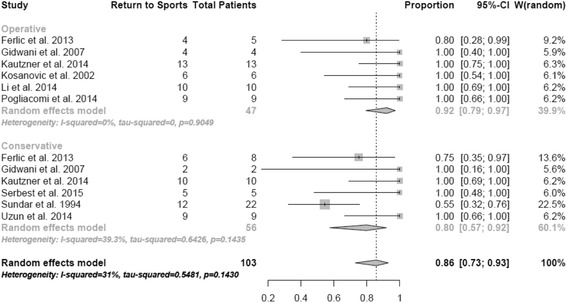
Return to sports at follow-up. Random effect model representing the proportion of athletes who returned to their preinjury activity-level. The horizontal lines extending out of the squares represent the 95% CI surrounding the best estimate. The diamond represents the weighted pool with confidence intervals

Return to sports on the preinjury level was evaluated in a total of 103 patients. The combined rate of return to sport of all patients by meta-analysis according to the random effects model was 86% (95% CI, 0.73-0.93). 80% of the conservative and 92% of the operative treated patients (*p =* 0,03) returned to sports at follow-up (Fig. 6). Three studies report the mean delay of return to sports with better results after operative therapy compared to conservative therapy (12.6 weeks versus 17.0 weeks).

## Discussion

Apophyseal fractures in sportive adolescents represent a particular challenge for the treating physician. By now, no evidence-based guideline exists for the ideal treatment method in these demanding patients. The aim of this systematic review with meta-analysis was therefore to summarize the existing evidence in the literature.

Summarizing the outcome of conservative and operative treatment by performing a meta-analysis the major finding of this study was that excellent outcome and successful return to sport is significantly more often achieved by surgical treatment with comparable complication rates of both treatment methods.

Apophyseal avulsion fractures of the pelvis are rare injuries that typically occur in the adolescent athlete [10, 28]. Awareness of these injuries increased over the past few years due to better musculoskeletal imaging techniques and a growing participation of adolescent athletes in competitive sporting activities [1]. However, the symptoms of the avulsion fractures still may be misinterpreted. Gidwani et al. [7] reported 8 cases of avulsion fractures of the ischial tuberosity which were misdiagnosed as a hamstring tear. The patients concerned required more extensive surgery as a consequence of this delay. Therefore, a correct and timely diagnosis is crucial to ensure adequate treatment. Radiography of the pelvis in at least two planes should be performed in the patients with typical clinical findings and an adequate history of trauma. In cases of unsuspicious radiographs, MRI or ultrasound can be used to reveal a potential soft tissue injury [5, 7]. Displacement of pelvic apophyseal fractures is restricted by the relatively thick periosteum and surrounding fascia in adolescents, thus nonoperative treatment with a guided rehabilitation program is considered the treatment of choice in undisplaced fractures [1, 3, 29].

Nonoperative treatment consisting of analgetics, limited activities, and partial weight bearing using crutches for at least 3–6 weeks has shown to be successful in many of these injuries [3, 21, 23, 25–27]. Metzmaker et al. presented successful nonoperative treatment of 27 avulsion fractures using a 5-phase rehabilitation protocol with full return to sports not until 2 months after injury [3]. This appears to be a relatively long period of reconvalescence that adversely disrupts the regular training for the young athletes [11]. Moreover, conservative treatment in some cases has a negative impact on short- and especially long term health in adolescent patients. Potential complications of conservative treatment include non-union or heterotopic ossifications and the “hamstring syndrome” in which shortening and fibrosis develop at the origin of the hamstrings after IT avulsion fractures [5, 26, 30]. These complications may be associated with chronic pain at the former fracture site and a significantly decreased ability to perform sports [8, 25]. Ferlic et al. reported the development of a pseudarthrosis in half of the conservatively treated patients with a displacement of the ischial tuberosity of more than 15 mm [5]. An excellent outcome could not be achieved in these patients.

In our study the complication rate was comparable (*p =* 0,47) in the conservative subgroup and the operative subgroup (17.0% vs. 19.0%). Kautzner et al. reported two patients with prolonged wound healing, three who developed keloid scars, one with pain and restricted movement who had to abandon his sports activity and two with heterotopic ossifications in their surgically treated group, consisting of 13 patients [22]. Actually none of these complications required additional surgical treatment; heterotopic ossifications in the surgically treated group were removed during screw extraction.

Due to these promising results, authors increasingly propose surgical treatment of apophyseal fractures [1, 5, 7, 10, 12, 22, 24, 31]. The results of the present study support these recommendations. Comparing the outcome of conservative and operative treatment by performing a meta-analysis, excellent outcome is achieved significantly more often after surgery, especially if fragment displacement is more than 15 mm. In these cases with significant fragment displacement and in patients with a high functional demand, surgery should be discussed as a treatment option.

### Limitations

This study has several limitations. As with all other reviews and meta-analyses, the quality is based on the quality of the studies analysed. Of the 14 studies included all of them were of Level 4 evidence and according to the results of the Coleman Methology Score the methological quality of the included studies was generally low. Prospective, randomized, controlled trials would be more suitable to answer the questions raised. Nevertheless, due to logistical concerns, especially the infrequency of apophyseal fractures, the studies of avulsion fractures are yet limited to retrospective analyses. However, to our knowledge, this study summarizes the largest amount of avulsion fractures ever reported in the literature. Additionally, return to sports may depend on more factors than a successful fracture consolidation and it may be argued that not all patients aimed to return to the preoperative activity level, thus influencing the study findings. In fact, the ability to return to sports may be higher than the rate of patients actually returning to sports.

## Conclusions

Both conservative and operative treatment provide satisfactory results in most of the apophyseal fractures. However, on the basis of the data recorded in the present meta-analysis, the overall success and return to sports rate was higher in the patients receiving surgery. Especially patients with fragment displacement greater 15 mm and high functional demands may benefit from surgical treatment. Families should be counseled accordingly.

## Abbreviations

AIIS: Anterior inferior iliac spine
ASIS: Anterior superior iliac spine
CMS: Coleman Methodology Score
IC: Iliac crest
IT: Ischial tuberosity
LT: Lesser trochanter
SCPS: Superior corner of the pubic symphysis
